# Complex and Controversial Roles of Eicosanoids in Fungal Pathogenesis

**DOI:** 10.3390/jof7040254

**Published:** 2021-03-28

**Authors:** Susana Ruiz Mendoza, Daniel Zamith-Miranda, Tamás Takács, Attila Gacser, Joshua D. Nosanchuk, Allan J. Guimarães

**Affiliations:** 1 Laboratório de Bioquímica e Imunologia das Micoses, Departamento de Microbiologia e Parasitologia, Instituto Biomédico, Universidade Federal Fluminense, Niterói 24210-130, RJ, Brazil; susa15_6@hotmail.com; 2 Departments of Medicine (Division of Infectious Diseases) and Microbiology and Immunology, Albert Einstein College of Medicine, Bronx, NY 10461, USA; danielzamith@gmail.com; 3 Department of Microbiology, University of Szeged, 6726 Szeged, Hungary; Takt@in1st.szote.u-szeged.hu (T.T.); gacsera@gmail.com (A.G.); 4 MTA-SZTE Lendület Mycobiome Research Group, University of Szeged, 6726 Szeged, Hungary

**Keywords:** eicosanoids, immune response, fungi, fungal eicosanoids, pathogenesis

## Abstract

The prevalence of fungal infections has increased in immunocompromised patients, leading to millions of deaths annually. Arachidonic acid (AA) metabolites, such as eicosanoids, play important roles in regulating innate and adaptative immune function, particularly since they can function as virulence factors enhancing fungal colonization and are produced by mammalian and lower eukaryotes, such as yeasts and other fungi (*Candida albicans*, *Histoplasma capsulatum* and *Cryptococcus neoformans*). *C. albicans* produces prostaglandins (PG), Leukotrienes (LT) and Resolvins (Rvs), whereas the first two have been well documented in *Cryptococcus* sp. and *H. capsulatum*. In this review, we cover the eicosanoids produced by the host and fungi during fungal infections. These fungal-derived PGs have immunomodulatory functions analogous to their mammalian counterparts. Prostaglandin E_2_ (PGE_2_) protects *C. albicans* and *C. parapsilosis* cells from the phagocytic and killing activity of macrophages. *H. capsulatum* PGs augment the fungal burden and host mortality rates in histoplasmosis. However, PGD_2_ potentiates the effects and production of LTB_4_, which is a very potent neutrophil chemoattractant that enhances host responses. Altogether, these data suggest that eicosanoids, mainly PGE_2_, may serve as a new potential target to combat diverse fungal infections.

## 1. Introduction

Fungal infections are a major global threat, particularly due to their increasing prevalence in immunocompromised patients [1], the limited number of therapeutic options, their chronicity, and frequently time-consuming diagnosis [2,3]. Classical virulence factors of pathogenic fungi include the presence of urease, proteases, heat shock proteins, melanins and a polysaccharidic capsule and other structures such as α-glucans and mannans, among many others, which contribute to the spread of the pathogens and modulation of host immune responses [4]. During fungal infections the role of inflammatory mediators such as cytokines, growth factors and chemokines has been widely studied, and these products have been considered the main soluble protein mediators of host defense against pathogens. However, the role of lipid mediators during fungal infections has not been fully explored and a variety of unique lipids can also play important roles in regulating innate and adaptive immune functions [5,6,7].

**Figure 1:**
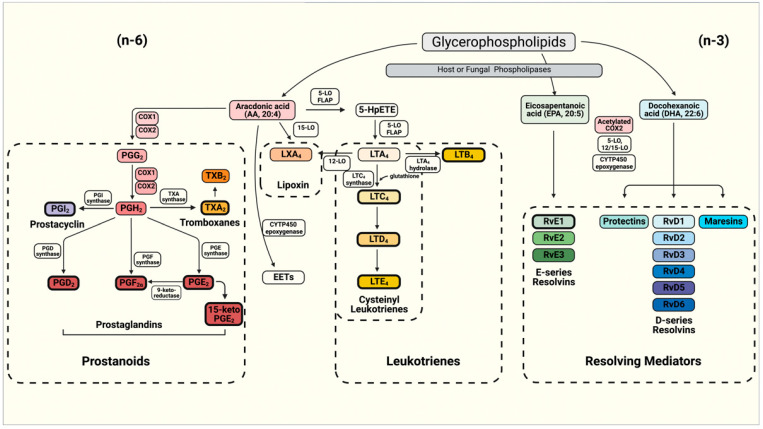
Schematics of the eicosanoids synthesis pathway for the production of prostanoids (Prostaglandins—PGs, Prostacyclin and Thromboxanes—TXs), Leukotrienes (LTs) and resolving mediators including D- and E-series resolvins (Rvs), protectins and maresins. The boxes depicted with bold borders illustrate the eicosanoids produced by fungi.

Biologically active lipid mediators derive from omega-3 (n-3) and omega-6 (n-6) polyunsaturated fatty acids (PUFA) [8] and include the 20-carbon arachidonic acid (AA; (20:4, n-6)) and eicosapentaenoic acid (EPA; (20:5, n-3))-derived eicosanoids and docosahexaenoic acid (DHA; 22:6(n-3))-derived docosanoids. These PUFAs, usually obtained from dietary sources or released from membrane phospholipids upon the hydrolysis of esterified fatty acids (FAs) by phospholipase A2 (PLA2), can be oxidized by three distinct main pathways involving cyclooxygenase (COX), lipoxygenase (LOX), and heme-containing cytochrome P450 (CYTP450) oxidase or epoxygenase enzymes (Figure 1) [8]. Classic *n*-6 PUFA AA-derived eicosanoids participate actively during immune responses [4,9], and can be classified into the prostanoids such as prostaglandins (PGs), prostacyclin (PGI_2_) and thromboxanes (TXs), in addition to leukotrienes (LTs) and lipoxins [10]. In contrast with lipoxins, which are formed from AA, the pro-resolving mediators (SPMs) such as protectins, resolvins (RVs) and maresins [11] have *n*-3 PUFAs as their precursors, i.e., EPA and DHA [12].

An important feature about AA-derived eicosanoids is their short response time, as their formation does not require protein synthesis, due to the fact that the AA precursor is present in mammalian cell membranes and the converting enzymes are usually constitutively expressed. However, these compounds can also be produced by lower eukaryotes, including yeasts and other fungi, having an active role during infection and representing a potential class of virulence factors [4,13].

**Prostaglandins (PGs)** are five-carbon ring eicosanoids that are produced through the conversion of AA to prostaglandin H_2_ (PGH_2_) by the cyclooxygenase-1 and -2 enzymes (prostaglandin endoperoxide H synthases COX-1 and COX-2, respectively) [5]. Depending on the following enzymatic step, PGH_2_ can be modified to produce different PGs (PGF_2α_, PGD_2_, and PGE_2_), prostacyclin (PGI_2_) or thromboxane A_2_ (TXA_2_) [14]. They regulate numerous processes throughout the body, such as kidney function, platelet aggregation, neurotransmitter release, and modulation of inflammatory responses, where they participate, among other tasks, in thermoregulation (inducing fever) and pain [5]. PGs bind to distinct types of GPCRs (G-protein coupled receptors), consisting of DP1 (Prostaglandin D_2_ receptor 1) and or CRTH2 (chemoattractant receptor-homologous molecule expressed on Th2 cells; also known as DP2, PG DP2 receptor) that recognize PGD_2_, rhodopsin-type receptors (EP1, EP2, EP3, EP4) that recognize PGE_2_, FP (prostaglandin F receptor) that recognizes PGF_2α_, IP (prostacyclin receptor) that recognizes PGI_2_, and TP (thromboxane receptor) that recognizes TXA_2_ [15,16,17,18]. These GPCRs generate several second messengers and trigger distinct signal transduction pathways [19]. EP1 induces intracellular Ca^2+^ mobilization via the G_q_ protein, whereas EP2 and EP4 increase cyclic adenosine monophosphate (cAMP) production via G_s_ and EP3 inhibits adenyl cyclase (thus decreasing cAMP) via G_i_ and elicits Ca^2+^ mobilization and phosphoinositide 3-kinase (PI3K) activation [15,20,21,22,23]. For these reasons, they modulate the activation of protein kinase A (PKA), transcription factors such as CREB [24], and extracellular signal-regulate kinases (ERKs) as well as the expression of cytokines during the immune response [13,25,26]. PGE_2_ is the most studied PG, which is produced by several cells such as macrophages and fibroblasts, and has diverse effects on the regulation and activity of distinct cells [5]. For example, PGE_2_ can modulate the activity of professional antigen presenting cells (APCs) such as dendritic cells (DCs) and macrophages and the production of cytokines [5]. 

Together with PGs in the prostanoid groups, **thromboxanes (TXs)** are produced as a six-member ether-containing ring upon the catalysis of the thromboxane synthase (TXS), producing the intermediate TXA_2_ or the final synthesis product TXB_2_ [10]. The thromboxane receptor (T prostanoid receptor, TP) is a GPCR, with either a G_q_ or G_12/13_ coupled subunit [10,27]. TXs are produced by several types of cells such as monocytes, macrophages, epithelial, and endothelial cells as well as platelets (thrombocytes), promoting the activation/aggregation and degranulation of platelets leading to the formation of blood clots [10,28,29]. TXA_2_ is the most potent known vasoconstrictor, and its proinflammatory action occurs by enhancing the activation of monocytes, cytokine production, expression of leukocyte adhesion molecule, and vascular permeability [29]. TXA_2_ also promotes T-cell activation and proliferation, and facilitates the development of effector cytolytic T-cells [7]. For instance, TXA_2_ participates in the damage caused by ischemic injury and inflammation in acute stages of *Trypanosoma cruzi* infections [29], exacerbates acute lung injury by promoting edema formation [27] and its excessive production causes significant hyper-permeability, resulting in severe edema by disrupting the endothelial barrier via Ca^2+^/Rho kinase signaling [30]. In addition to these immunomodulatory functions, TXs receptors (TPs) are expressed in high levels in the thymus where they participate in the negative selection of maturing T lymphocytes [7,30].

**Leukotrienes (LTs)** are synthetized from AA by the enzyme 5-lipoxygenase (5-LO) and 5-lipoxygenase activating protein (FLAP) into 5-hydroperoxyeicosatetraenoic acid (5- HpETE), which is further metabolized into leukotriene A_4_ (LTA_4_), the precursor of all forms of LTs [31]. LTA_4_ is converted by LTA_4_ hydrolase (LTA4H) into leukotriene B_4_ (LTB_4_), or it can be conjugated with reduced glutathione by leukotriene C_4_ (LTC_4_) synthase to yield the cysteinyl leukotriene (CysLT) LTC_4_ and its derivatives [31]. LTB_4_ and LTC_4_ are exported via the specific ATP-binding cassette (ABC) transporters-1 and -4, whereas further released LTC_4_ is converted to leukotriene D_4_ (LTD_4_), which can undergo further conversion into leukotriene E_4_ (LTE_4_) [31,32]. LT receptors are also GPCRs located on the outer plasma membrane of resident and inflammatory cells, among other cell types. They induce the increase in intracellular Ca^2+^ and the reduction in intracellular cAMP levels [31,33,34]. LTB_4_ binds to BLT1 and BLT2 receptors, whereas the most known receptor of cysteinyl LTs is the type 1 CysLT receptor (CysLTR1), with high affinity for LTD4 and it is the target for antagonists clinically used for the management of asthma, such as Montelukast, Zafirlukast and Pranlukast [31,32,34,35]. LTs play an important role in amplifying the inflammatory responses to infection [31]. LTB_4_ participates in the activation and recruitment of neutrophils, macrophages, monocytes, mast cells, and T lymphocytes, while increasing phagocytosis, microbicidal activity, and generating and modulating chemokines and cytokines [31]. It is one of the main modulators of the activation and maintenance of the innate and adaptive immune response [35,36]. Fungal zymosan and peptidoglycan from *Aspergillus fumigatus* induce the production of LTs in the airways that contributes to the initiation of asthma and causes and exacerbates potent bronchoconstrictive effects, such as edema through vasodilation, increased vascular permeability, and enhanced recruitment of effector cells [37]. In contrast, gliotoxin from *A. fumigatus* suppresses the biosynthesis of LTB_4_ by direct interference with LTA4H activity resulting in impaired neutrophil functions [38,39,40]. 

Non-classical eicosanoids compose the group of specialized pro-resolving mediators (SPM) also called **resolvins (Rvs)** [8]. SPMs derived from EPA are designated E-series Rvs (Resolvin E1 or RvE1, RvE2 and RvE3), whereas those from DHA are referred to as D-series RVs (RvD1-6) [12,41] (Figure 1). Four further metabolites of DHA have a hydroxyl group at the 13-position and have been designated as 13-series resolvins (RvT). DHA is converted to three Rvs of which RvD1(n-3DPA) is the most abundant [8]. RVs are involved in the resolution stage of inflammation, ending the chronicity of the inflammatory process and, hence, reducing or preventing tissue damage [11,12]. RvE1 is an eicosanoid that protects human tissues from leukocyte regulated inflammatory processes [42,43,44]. RvE1 dramatically reduces dermal inflammation, peritonitis and interleukin (IL) production and inflammatory pain [45]. RvE2 can effectively reduce joint pain in arthritis [11]. RvD2 ameliorates bacterial sepsis, with RvD3 acting in later stages of resolution and RvD4 helping the clearance of apoptotic cells by skin fibroblasts [8]. In general, RvDs also block tumor necrosis factor (TNF)-α-induced IL-1β transcripts and are potent regulators of PMN infiltration in brain, skin, and peritonitis in vivo [11,12].

## 2. Molecular Basis of Eicosanoid Production in Fungi

**Figure 2:**
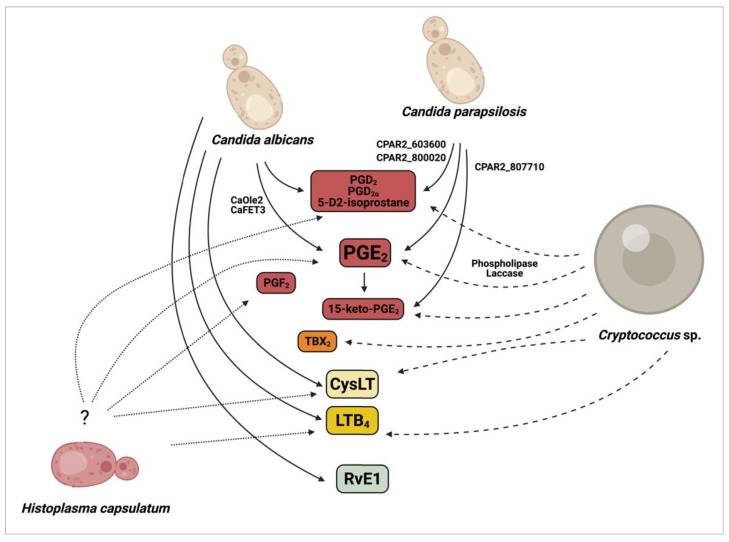
Eicosanoids production in *Candida* sp., *Cryptococcus* sp. and *Histoplasma capsulatum*. The figure illustrates genes involved in the synthesis of eicosanoids, with exception of *H. capsulatum*, with as yet undescribed genes involved. Lines and arrows indicate the eicosanoids produced by *Candida* sp. (solid lines), *Cryptococcus* sp. (dashed lines) and *H. capsulatum* (dotted lines).

The molecular background of eicosanoid biosynthesis was first revealed in mammals, with the description of three main enzymes pathways (COX, LO, and CYTP450) [46]. Eicosanoid production in yeasts was first uncovered in the early 1990′s in the non-pathogenic fungus *Dipodascopsis uninucleata*. Van Dyk and colleagues isolated a 20-carbon chained AA metabolite identified as 3-hydroxy-5,8,11,14- eicosatetraenoic acid (3-HETE) [47]. Later, the same oxylipid was found in other yeasts of *Dipodascaceae* spp. and the filamentous *Mucor* spp. and *Rhizomucor* spp. [48,49]. Noverr et al. [13] examined several pathogenic fungi for the production of eicosanoids, and each analyzed species was able to produce compounds that eluted together with mammalian PGs and LTs, in the absence and presence of exogenous AA, by either, respectively de novo or a “*trans*-species” mechanism with fungal phospholipases acting on host phospholipids (Figure 2) [6].

However, whole genome sequencing analyses revealed that fungi have no homologues for the abovementioned mammalian enzymes, suggesting that fungi have evolved alternative routes for the synthesis of eicosanoids [46]. Yet, the use of COX inhibitors, such as aspirin, indomethacin, and etodolac and the inhibition of the LO pathway with nordihydroguaiaretic acid inhibited eicosanoids production and clearly impacted growth of *Cryptococcus neoformans* and *Candida albicans*, offering a link between fungal growth and eicosanoid production [50,51,52].

### 2.1. Production of Eicosanoids by Candida albicans and Non-Albicans Species

Deva et al. revealed that the opportunistic human fungal pathogen *C. albicans* produces 3,18-dihydroxy-5,8,11,14- eicosatetraenoic acid (3,18-di-HETE) by utilizing exogenous AA [53]. A subsequent study reported that, besides 3,18-di-HETE, *C. albicans* synthesizes an uncharacterized prostaglandin (PGE_x_) [50]. This eicosanoid was later shown to be indistinguishable from mammalian PGE_2_ [52]. Further investigations identified two non-COX/LO/CYTP450-related enzymes, namely the fatty acid stearyl-coenzyme A desaturase (Ole2) and the multicopper ferroxidase (Fet3), which are potentially involved in *C. albicans* (Ca) PGE_2_ biosynthesis (Figure 2) [13,52]. Homozygous deletion of both the fatty acid desaturase CaOLE2 and the multicopper oxidase CaFET3 resulted in a significant reduction in PGE_2_ synthesis by approximately 50–70% and 40–50%, respectively. However, PGE_2_ levels were still measurable in the corresponding homozygous mutant suggesting the presence of yet undiscovered PGs regulatory pathways in this species. *C. albicans* is also able to produce other PGs, such as PGD_2_ and PGD_2α_ [13,52].

Besides *C. albicans*, several non-*albicans Candida* species such as *C. dubliniensis*, *C. tropicalis*, *C. glabrata,* and *C. parapsilosis* synthesize PGE_2_ [54,55,56], all of which are frequently associated with human fungal infections. HPLC-MS analysis of the fatty acid biosynthesis of *C. parapsilosis* by Grózer and colleagues revealed that this species, similar to *C. albicans*, is able to produce various PGs besides PGE_2_, and highlighted PGD_2_ as another major eicosanoid produced by *C. parapsilosis* [56]. A 2018 follow-up study with *C. parapsilosis* also identified an uncommon oxylipin, an autoxidative isomer of PGD_2_ (5-D2-IsoProstane) secreted upon incubation with exogenous AA (Figure 2) [57].

However, our knowledge of its biosynthesis is scarce [58]. A recently published study by Chakraborty and colleagues aimed to identify the molecular basis of PG production in *C. parapsilosis* and identified several genes involved in the process [57]. These include CPAR2_603600 (a homologue of the CaFET3), CPAR2_807710 (Acyl-CoA oxidase in *S. cerevisiae*, ScPOX1-3) and CPAR2_800020 (Acyl-CoA thiolase in *S. cerevisiae*, ScPOT1) (Figure 2). LC/MS data revealed that *C. parapsilosis’* PGE_2_ biosynthesis is decreased by approximately 60–70% if any of these genes are disrupted. The double deletion of CPAR2_603600 and CPAR2_800020 leads to about 80% decrease in PGD2 production, suggesting their significant role in its biosynthesis. Their removal also effected the secretion of 15-keto-PGE_2_, a metabolite generated by the degradation of PGE2. CPAR2_807710 was shown to be most involved in 15-keto-PGE_2_ production. In contrast to *C. albicans*, the homologue of CaOLE2 has no significant role in PGE_2_ biosynthesis in *C. parapsilosis* [56].

Notably, in addition to PGs, *C. albicans* also utilizes AA for the biosynthesis of LTs, such as LTB_4_ and CysLTs (Figure 2) [13]. During *Candida* spp. infection, the synthesis of some LTs is altered to reduce host immune responses as a strategy for the establishment and maintenance of the infection [35]. LTB_4_ and CysLT production are both mediated by lipoxygenases through the production of 5-HpETE from exogenous AA [13], whereas RvE1 synthesis in *C. albicans* is produced from EPA [42], and some biosynthetic precursors (18-HEPE, 15-HEPE and 5-HEPE), by neutrophil 5-lipoxygenase principally, cytochrome P450 monooxygenase enzymes (CYP45), and other specific enzymes remain unknown [13,42,59]. The detailed biosynthetic pathway of LTs and RvE1 in *C. albicans* also remains enigmatic. Other human pathogenic non-*albicans Candida* species such as *C. dubliniensis*, *C. tropicalis*, and *C. glabrata* may also be able to produce these eicosanoids; however, this remains unconfirmed.

### 2.2. Production of Eicosanoids by Cryptococcus sp.

*C. neoformans* produces biologically active eicosanoids from exogenous sources of AA during infection, which are indistinguishable from host eicosanoids and modulate host defenses [50,51]. The major AA metabolite produced is an authentic PGD_2_, but the fungus is also able to produce heptadecatrienoic acid, 5-HETE, PGF_2_, TXB_2_, and PGE_2_ [50]. Two enzymes expressed by *C. neoformans*, phospholipase B1 (PLB1) and laccase (CNLAC1 gene), are believed to be associated with cryptococcal eicosanoid synthesis (Figure 2). Pharmacological enzymatic inhibition or deletion of phospholipase B1 (Δ*plb1*) reduces secreted levels of all eicosanoids produced by *C*. *neoformans* [60,61]. In turn, deletion of laccase (Δ*lac1* mutants) or enzymatic inhibition by anti-lac1 antibody resulted specifically in the loss of PGE_2_ [51]. The addition of PGE_2_ was sufficient to promote growth of Δ*plb1* and Δ*lac1* in vitro and in vivo, independently of host PGE_2_ [60,61]. In fact, laccase is an important virulence factor for *C. neoformans* with a broad spectrum oxidase activity, converting polyphenolic compounds into the cell wall pigment melanin, and this polymer protects *C. neoformans* against oxidants, microbiocidal proteins and antifungals as well as to phagocytosis and killing by macrophages [62,63]. Additionally, recombinant laccase readily converts PGG_2_ into PGE_2_ and 15-keto-PGE_2_, and it is suggested as a key cryptococcal prostaglandin enzyme for this recently described unique production pathway (Figure 2) [51].

### 2.3. Production of Eicosanoids by Histoplasma Capsulatum

Although *Histoplasma capsulatum* can produce eicosanoids [13,54], further studies are necessary to dissect the pathways involved in their production and to determine whether they play a role during infection (Figure 2).

## 3. The Role of Eicosanoids during Fungal Infections

The production of eicosanoids by pathogenic fungi, such as *C. albicans, C. dubliniensis, C. glabrata, C. tropicalis, C. neoformans, H. capsulatum* and *A. fumigatus* is linked to the pathogenesis of each fungal infection [4,9,51,60,64,65,66]. Some fungal-derived eicosanoids can enhance both fungal colonization and induce immunomodulatory effects. Overall, fungal LTs act by enhancing the acute inflammation, whereas PGs have negative effects on innate and cellular Th1 responses against mycosis, resulting in immunological tolerance and contributing to the chronicity of fungal infections [13]. Herein, we discuss the roles of eicosanoids in three major fungal infections.

### 3.1. Eicosanoids in Candidiasis

Eicosanoids play an important role in both sides of the host–*Candida* interaction. Depending on the organ or tissue environment, host-derived PGE_2_ either decreases [64,67] or improves [68,69] the protective Th1 and Th17 responses that particularly may help the host restrain *C. albicans* at barrier surfaces and in the bloodstream.

*C. albicans* induces host cells to release AA from membrane phospholipids and infection-derived stimuli can also induce COX-2 expression and trigger the synthesis of PGs in various cells types [66,70]. *C. albicans* stimulates AA metabolism and the generation of PGE_2_ by synovial fibroblast, alveolar and peritoneal macrophages, and epithelial cells via stimulation of TLR2 and TLR4 [14]. *Candida* mannans and β-1,3-glucan induce PGE_2_ via stimulation of mannose receptor and dectin-1 in peripheral blood mononuclear cells, respectively [71]. PGE_2_ signaling stimulates Th2 and Th17 responses to yeast and limits the ability of macrophages to clear *Candida* sp. [71].

Although the exact role of *Candida*-derived eicosanoids during host–pathogen interactions is largely undiscovered, a limited number of studies are available that provide insights into how these lipid metabolites affect fungal virulence [57,67]. Many studies have pointed out the major role of host derived AA and fungi derived PGE_2_ in the modulation of yeast cell growth, morphogenesis, and biofilm formation in *C. albicans* [50,55]. In contrast, some studies focusing on the negative impact of PGE_2_ on yeast biology have shown that PGE_2_ inhibits germ tube formation by antagonizing yeast to hyphal transformation in *C. albicans*, which may limit tissue invasion [72].

In a previous study, the PGE_2_ biosynthesis associated genes OLE2, FET3, and FET31 were knocked out in *C. albicans* strains and the mutant’s capacity for PGE_2_ secretion was decreased in vitro. The authors examined the killing of the mutants by macrophages and immune-modulatory effects in vitro as well as their capacity for organ colonization ability in various mouse models of invasive candidiasis. The *ole2^−/−^* showed similar fitness and rates of hyphal formation than the wild-type (WT) counterpart. However, the gut colonizing capacity of the *ole2^−/−^* strain decreased compared to the WT strain. Besides its role in promoting colonization and survival in the mouse gut, *C. albicans* derived PGE_2_ also inhibited fungal cell internalization by phagocytes [65]. However, in CD11b+ DC and macrophage depleted mice, the WT *C. albicans* strain was not able to overgrow the ole2^−/−^ strain [65], suggesting that the presence of PGE_2_ is beneficial for fungal growth, overcoming phagocytosis, and enhancing survival within the host.

Regarding non-*albicans Candida* species, the presence of AA increases biofilm formation and PGE_2_ production by *C. glabrata*, *C. parapsilosis,* and *C. tropicalis* [58]. These findings suggest that *Candida spp*. evolved the capacity to produce PGs, primarily PGE_2_, to enhance their fitness and survival within certain niches of the host that could directly promote the fungus’ pathogenesis upon a potential commensal-to-pathogenic shift event. The work of Chakraborty and colleagues suggests that fungal-derived PGs in *C. parapsilosis* also negatively regulate yeast cell phagocytosis and killing by macrophages, as PGs (PGE_2_, PGD_2_, and 15-keto-PGE_2_)-deficient *C. parapsilosis* cells were more susceptible to phagocytosis and killing by human peripheral blood monocyte-derived macrophages (PBMC-DM) compared to the WT strain [57]. As the virulence of PG deficient *C. parapsilosis* mutant strains also decreased in vivo compared to the WT strain, fungal PGs could also actively contribute to the virulence of this species.

These observations, together with other previous reports, suggest that fungi-derived prostaglandins have immunomodulatory functions analogous to their mammalian counterparts [54,73]. To further support this suggestion, another study reported that *C. albicans*-produced PGE_2_ up-regulates anti-inflammatory responses through enhancing IL-10 released by murine splenocytes. Moreover, the levels of mouse keratinocyte-derived chemokine (KC, analog to human IL-8) and other pro-inflammatory cytokines, such as TNFα, decreased after 24 h of fungal PGE_2_ treatment [67,71]. Fungal-derived PGE_2_ decreases the killing of *C. albicans* by intestinal macrophages, supporting the idea that fungal prostaglandins could also inhibit the killing activity of host cells.

A similar conclusion can be drawn for *C. parapsilosis*, where the absence of PGE2 -related genes increased the expression of pro-inflammatory cytokines such as pro-IL-1β, IL-6 and TNFα [57]. Thus, *C. parapsilosis* PGE_2_ could also negatively regulate host inflammatory responses. In *C. parapsilosis*, PGs production actively contributes to host cell damage, as revealed by the decreased [57] death of PBMC-DMs following infection with PG-deficient strains compared to the WT strain. *C. parapsilosis* PGs secretion is also suggested to contribute to organ colonization when studied in a mouse model of systemic candidiasis. However, the studied PG-related genes contributed unequally to the fungal load of each examined organ, which may suggest that the observed effect is not solely due to the presence of fungal PGE_2_, PGD_2_ and 15-keto-PGE_2_ [57].

LTs were also described as biologically active immunomodulatory eicosanoids [74,75]. Host-derived LTs increase capillary permeability, and activate and recruit eosinophils and neutrophils [75]. The present literature lacks information about the immunomodulatory function of fungal-derived LTs. However, a recent study showed that the amount of LTF4 increased in patients with candidemia, suggesting that LTF4 may also contribute to host responses to *Candida* spp. [35].

A previous study in 2007 showed that *C. albicans*-derived RvE1 is chemically identical to the human RvE1 [42]. When administered at low concentrations, fungal RvE1 reduced the IL-8-mediated chemotaxis of human neutrophils and also the recruitment of DCs [42,59]. In contrast, higher doses of fungal RvE1 enhanced phagocytic activity and fungicidal reactive oxygen species (ROS) production by human neutrophils against *C. albicans*. Interestingly, inoculation of RvE1 into mice with fungemia due to *C. albicans*, led to a more rapid clearance of the pathogen from the bloodstream [42]. These facts suggest that low concentrations of fungal RvE1 protects *C. albicans* due to the inhibition of neutrophil recruitment, although higher fungal burden (together with increased fungal RvE1 levels) could act as an alarming signal for neutrophils, which would then be able to control and restrict fungal invasion.

### 3.2. Eicosanoids in Cryptococcosis

*C. neoformans* secretes phospholipase B (PLB), which is a virulence factor. This single cryptococcal protein has three separate enzymatic activities: phospholipase B (PLB), which removes both acyl chains simultaneously from phospholipids; lysophospholipase (LPL), which removes the single acyl chain from lysophospholipids; and lysophospholipase transacylase (LPTA), which adds an acyl chain to lysophospholipids to form phospholipids [61]. Despite the lack of understanding on the structure and mechanism of action of PLB, this enzyme is involved in the survival of *Cryptococci* within macrophages, the destruction of lung tissue and the production of eicosanoids, which modulate phagocytic activity [61]. As mentioned, *C. neoformans* produces eicosanoids from exogenous AA and utilizes them to modulate the immune response favoring its own survival. For instance, LTB_4_ significantly reduced neutrophil recruitment in the lung vasculature of mice infected intravenously with *C. neoformans*, demonstrating a critical role of LTB_4_ in intravascular neutrophil swarming during infection [76]. The presence of CysLTs and LTB_4_ produced by *C. neoformans* strains B-3501A and H99 through the activity cryptococcal phospholipase cPLA2α and 5-LO, can contribute to fungal penetration of the blood–brain barrier in vitro and in vivo, specifically facilitating central nervous system (CNS) infection [77].

*C. neoformans* is also able to modulate the host inflammatory state during infection by directly manipulating host eicosanoids signaling and PGE_2_ is considered a mediator of cryptococcal virulence [60,78]. During macrophages infection, *C. neoformans* produces the dehydrogenated form of PGE_2_ (15-keto-PGE_2_) enhancing its virulence via the activation of the host nuclear transcription factor, PPAR-γ [60]. In *C. neoformans* infections, the use of antagonists of either EP2 or EP4 receptors improves the host defense by promoting TLR-4-mediated cytokine production, and enhancing M1 macrophage polarization followed by yeast killing [78].

### 3.3. Eicosanoids in Histoplasmosis

A 1992 study showed that peritoneal macrophages challenged with heat-killed *H. capsulatum* produce prostanoids (PGE_2_ and PGI_2_) and LTs (LTB_4_ and LTC_4_), the former being produced in a COX-dependent fashion [79]. This first observation was the stepping-stone for the study of eicosanoids in histoplasmosis. Notably, different forms of LTs and PGs are produced by the host during in vitro and in vivo challenges with *H. capsulatum*, but, interestingly, they commonly have opposite roles [80,81].

Sub-lethal *H. capsulatum* infections in mice treated with a FLAP inhibitor or in 5-LO deficient mice are fatal, suggesting that LTs are important for the host response in histoplasmosis [81,82]. Even though LTB_4_ and LTC_4_ are produced in mice infected with *H. capsulatum* [81], data show that administration of microspheres-associated LTB4 to 5-LO deficient mice can restore the production of cytokines and control the fungal burden [83]. 

Although LTB4 is an important mediator for the host response against *H. capsulatum*, the mechanism behind its effects is controversial. LTB_4_ is a very potent neutrophil chemoattractant [84], but 5-LO deficient mice and mice treated with FLAP inhibitors have lower levels of LTs and increased neutrophil recruitment when compared to their control counterparts. The increased neutrophil recruitment is followed by higher inflammatory response, an elevation of splenic fungal burdens, and 100% of mortality 14 days post-infection, even in scenarios of non-lethal *H. capsulatum* infections [81,82]. This suggests that features other than neutrophil chemotaxis are behind LTs’ effects during histoplasmosis. The effector mechanisms employed by macrophages are also responsive to LTs, as 5-LO deficient mice have a remarkable impairment in their ability to phagocytose non-opsonized or even IgG-opsonized *H. capsulatum* yeast cells, a deficiency that is bypassed by the exogenous addition of LTB_4_ or LTC_4_ [82]. Although LTs as well as PGs are usually produced at the onset of the inflammatory process, further steps in the host defense are modulated by the presence of these mediators [85]. Immunization of mice with cell-free antigens from *H. capsulatum* fails to confer protection in 5-LO deficient mice, possibly due to an inability to induce the recruitment of CD4+ and CD8+ cells to the lungs, and also a failure to increase the production of IFN-γ [86]. The production of LTs has an impact on events of the innate, but also of the adaptive, response during *H. capsulatum* infection, which modifies the outcome of the host–pathogen interaction.

The role of PGs during *H. capsulatum* infection is not as well studied relative to the leukotrienes. A fundamental piece of data is that the inhibition of COX-2 protects mice against lethal infection with *H. capsulatum*, a phenotype marked by lower fungal burden and a milder inflammatory process [80]. Curiously, when inhibiting the synthesis of prostanoids, an increase in the synthesis of LTB_4_ is observed, which is also beneficial to the host. The higher survival rates are associated with a decrease in neutrophil recruitment, consistent with the effects of LTs [80]. PGE_2_ has been associated with the deleterious effects on *H. capsulatum* infection [16], which correlates with the expression and activity of galectin-1 (Gal-1) [87]. Gal-1 represses the expression of PGE_2_ synthase, thus reducing the levels of PGE_2_ in *H. capsulatum*-infected mice. In contrast, *H. capsulatum* infection in Gal-1 KO mice leads to an increase in PGE_2_ production followed by increased fungal burden and higher mortality rates when compared to WT mice [87]. Even though PGE_2_ has such deleterious effects to the infected host, PGD_2_ has opposite effects to PGE_2_. The pharmacological inhibition of the endogenous production of PGD_2_ in *H. capsulatum*-infected macrophages leads to a severe inhibition of the leukocyte’s fungicidal activity, an effect that is reversed by the exogenous addition of PGD_2_. PGD_2_ also upregulates the expression of LTB_4_ receptor (BLT1R), potentiating the effects of LTB_4_ [87]. The role and mechanism of eicosanoids in the host response against *H. capsulatum* is still understudied, but data suggest that LTB_4_ and PGE_2_ have opposite effects in histoplasmosis by modulating the recruitment of neutrophils and the effector mechanisms of macrophages. In agreement, PGE_2_ also inhibits the production of hydrogen peroxide and TNF-α by monocytes, limiting the killing of *Paracoccidioides brasiliensis* [88]. Further studies are necessary to dissect whether other eicosanoids have a role in the infection by *H. capsulatum*, including ones of fungal origin and also the mechanisms involved in immune regulation.

## 4. Concluding Remarks

Human pathogenic fungal species such as *Candida spp.*, *C. neoformans* and *H. capsulatum* produce eicosanoids. *C. albicans* utilizes exogenous AA in order to produce 3,18-di HET, LTB_4_, Cys-LTs, RvE1 and prostaglandins such as PGE_2_, PGD_2_, PGF2_α_. Non-*albicans Candida* species such as *C. dubliniensis, C. tropicalis, C. glabrata,* and *C. parapsilosis* also synthesize PGE_2_ from AA. Additionally, *C. parapsilosis* produces other prostaglandins such as PGD2 and 15-keto-PGE_2_. The exact molecular mechanisms behind the *Candida*-derived eicosanoid production are only uncovered in the case of PGs in *C. albicans and C. parapsilosis*. PGE₂ synthesis in *C. albicans* is regulated by OLE2, while *C. parapsilosis* evolved OLE2-independent PGs production pathways. This difference may explain the contrast in in vivo results: *C. albicans*-derived PGE_2_ is not required for virulence while PGs produced by *C. parapsilosis* influence the yeast’s capacity for host damage. Overall, the presence of fungal PGE_2_ has proven to be beneficial for *C. albicans* through increasing the ability of the pathogen to colonize the gut. Furthermore, fungal PGE_2_ protects *C. albicans* and *C. parapsilosis* cells from the phagocytic and killing activity of macrophages. *C. albicans*- derived RvE1 protects the fungus at low concentrations, whereas high concentrations expose the fungus to the host. *C. neoformans* produces 15-Keto-PGE_2_ to enhance its growth and ability to survive macrophage infection. In histoplasmosis, the inhibition of the PGs production is beneficial to the host as it favors LTB_4_ production, which induces a decrease in the fungal burden, mortality rates and neutrophil recruitment. PGE_2_ has deleterious effects on histoplasmosis, as opposed to the positive effects of PGD_2_, which upregulates the expression of BLT1R in *H. capsulatum* infected macrophages and potentiates the effects of LTB_4_. LTs are important for the host response, as, for example, LTB_4_ mediates the immune response helping to control the fungal burden. However, the mechanism behind its effect is controversial as LTB_4_ is a neutrophil chemoattractant and mice with lower levels of LTs have increased inflammatory responses, fungal burdens and mortality rates. However, further investigations are needed to understand the precise role of eicosanoids, mainly PGE_2_, during host–pathogen interactions.

## 5. Future Trends

The production of eicosanoids seems to be a conserved feature among several eukaryotic organisms, including filamentous and yeast fungi, protozoa and higher eukaryotes such as mammals. Independently on the organism, their biosynthetic pathways may vary considerably, as well as the full eicosanoid portfolio produced. Since pathogenic fungi are able to secrete these molecules, the exact mechanism in how they alter the microbial physiology has not been fully explored, although current research and published data have demonstrated their effects on the modulation of interactions with the host and immune responses.

Then, as it is completely plausible that fungal eicosanoids might function as virulence factors, further investigations might enable us to understand their precise role during host–pathogen interactions, as well as exploring the unique steps of the fungal eicosanoids biosynthesis, as a new potential target to combat *C. albicans*, *C. parapsilosis, C. neoformans* and *H. capsulatum* and possibly other fungal infections.

## Abbreviations

15-keto-PGE2	Dehydrogenated form of Prostaglandin E_2_
3-HETE	3-hydroxy-5,8,11,14- eicosatetraenoic acid
3,18-di-HETE	3,18-dihydroxy-5,8,11,14- eicosatetraenoic acid
5- HpETE	5-hydroperoxyeicosatetraenoic acid
5-LO	Enzyme 5-lipoxygenase
AA	Arachidonic acid
AA (20:4, n-6)	Arachidonic acid (20-carbon, 4 insaturations, omega 6 family)
ABC	ATP-binding cassette transporter
APCs	Antigen presenting cells
BLT1	Leukotriene B4 high-affinity receptor
BLT2	Leukotriene B4 Low-affinity receptor
CaFET3	*Candida albicans* multicopper ferroxidase
cAMP	Cyclic adenosine monophosphate
CaOLE2	*C. albicans* fatty acid stearyl-coenzyme A desaturase
CNLAC1	*Cryptococcus neoformans* laccase gene
CNS	Central nervous system
COX	Cyclooxygenase
CRTH2	Chemoattractant receptor-homologous molecule expressed on Th2 cells; also known as DP2, PG DP2 receptor
CysLT	Cysteinyl leukotriene
CysLTR1	Type 1 cysteinyl leukotriene receptor
CYTP450	Cytochrome P450 oxidase
DCs	Dendritic cells
DHA	Docosahexaenoic acid
DP1	Prostaglandin D_2_ receptor 1
EP (1-4)	Rhodopsin-type receptors
EPA	Eicosapentaenoic acid
ERKs	Extracellular signal-regulate kinases
FAs	Fatty acids
FLA	5-lipoxygenase activating protein
FP	Prostaglandin F receptor
Gal-1	Galectin-1
GPCRs	G-protein coupled receptors
IL	Interleukin
IP	Prostacyclin receptor
LOX	Lipoxygenase
LPL	Lysophospholipase
LPTA	Lysophospholipase transacylase
LT	Leukotriene
LTA4	Leukotriene A_4_
LTA4H	LTA_4_ hydrolase
LTB4	Leukotriene B_4_
LTD4	Leukotriene D_4_
LTE4	Leukotriene E_4_
LTF4	Leukotriene F_4_
LTs	Leukotrienes
n-3	Omega-3
n-6	Omega-6
PBMC- DM	Peripheral blood monocyte-derived macrophages
PGs	Prostaglandins
PGD_2_	Prostaglandin D_2_
PGE_2_	Prostaglandin E_2_
PGE_x_	Uncharacterized prostaglandin
PGF_2_	Prostaglandin F_2_
PGG_2_	Prostaglandin G_2_
PGH_2_	Prostaglandin H_2_
PGI_2_	Prostacyclin
PI3K	Phosphoinositide 3-kinase
PKA	Protein kinase A
PLA	Phospholipase A
PLB	Phospholipase B
PMN	Polymorphonuclear neutrophil
PUFA	Polyunsaturated fatty acids
Rvs	Resolvins
RvD	D-series Resolvins (RvD1-6)
RvE1	E-series Resolvins (RvE1-3)
RvT	13-series resolvins
SPM	Specialized pro-resolving mediator
Th	T-helper
TNF-α	Tumor necrosis factor alpha
TP	Thromboxane receptor
TX	Thromboxane
TXA2	Thromboxane A2
TXS	Thromboxane synthase
Δlac1	Laccase gene *Cryptococcus neoformans* mutant
Δplb1	Phospholipase B1 *Cryptococcus neoformans* mutant
